# Psychometric validation of the Moroccan version of the EORTC QLQ-C30 in colorectal Cancer patients: cross-sectional study and systematic literature review

**DOI:** 10.1186/s12885-021-07793-w

**Published:** 2021-01-27

**Authors:** Yacir El Alami, Hajar Essangri, Mohammed Anass Majbar, Saber Boutayeb, Said Benamr, Hadj Omar El Malki, Amine Souadka

**Affiliations:** 1 The Surgical Department, National Institute of Oncology, Rabat, Morocco; 2 Faculty Of Medicine Of Rabat, Mohammed V University, Rabat, Morocco; 3 The Doctoral School of Life and Health Sciences, Mohammed V University, (CEDOC SVS), Rabat, Morocco; 4 The Surgical Department ‘A’, Ibn Sina Hospital, Rabat, Morocco; 5 The Laboratory of Clinical Research and Epidemiology of Mohammed V University, Rabat, Morocco; 6 Abulcasis International University of Health Sciences, Rabat, Morocco

**Keywords:** *EORTC QLQ-C30*, Rectal neoplasm, Colonic neoplasms patient-reported outcome, Validation, Morocco

## Abstract

**Background:**

Health-related quality of life is mainly impacted by colorectal cancer which justified the major importance addressed to the development and validation of assessment questionnaires. We aimed to assess the validity and reliability of the Moroccan Arabic Dialectal version of the European Organization for Research and Treatment of Cancer (EORTC) Quality of Life Core Questionnaire (QLQ-C30) in patients with colorectal cancer.

**Methods:**

We conducted a cross-sectional study using the Moroccan version of the EORTC QLQ-C30 on colorectal cancer patients from the National Oncology Institute of Rabat, in the period from February 2015 to June 2017. The QLQ-C30 was administered to 120 patients. Statistical analysis included reliability, convergent, and discriminant validity as well as known-groups comparisons.

**Results:**

In total, 120 patients with colorectal cancer were included in the study with 38 (32%) patients diagnosed with colon cancers. Eighty-two patients (68%) had rectal cancer, among which 29 (24%) patients with a stoma. The mean age of diagnosis was 54 years (+/− 13.3). The reliability and validity of the Arabic dialectal Moroccan version of the EORTC QLQ-C30 were satisfactory. [Cronbach’s alpha (α =0.74)]. All items accomplished the criteria for convergent and discriminant validity except for question number 5, which did not complete the minimum required correlation with its own scale (physical functioning). Patients with rectal cancer presented with bad Global health status and quality of life **(GHS/QOL)**, emotional functioning as well as higher fatigue symptoms compared to patients with colon cancer. The difference between patients with and without stoma was significant for diarrhea and financial difficulty.

**Conclusions:**

The Moroccan Arabic Dialectal version of the QLQ-C30 is a valid and reliable measure of health-related quality of life **(HRQOL)** in patients with colorectal cancer.

**Supplementary Information:**

The online version contains supplementary material available at 10.1186/s12885-021-07793-w.

## Introduction

In the past decades, the advancement of disease management provided an impetus for the development of patient-reported outcomes, especially for cancer patients [1]. The traditional outcome measures, although meticulous when relying on examination maneuvers, laboratory tests, and imaging modalities, provide little correlation to the impact on the quality of life. A progression-free and improved overall survival is often not correlated to a satisfying life [2]. All these tendencies ushered many emerging terms such as health-related quality of life (HRQOL), defined as “those aspects of self-perceived well-being that are related to or affected by the presence of disease or treatment” [3].

Quality of life understanding led to many assessment tools with disease-specific applications, namely the QLQ C-30 questionnaire by the European Organization for Research and Treatment of Cancer (EORTC). The latter was updated into the currently used third version, which has been translated into more than 48 languages as well as transcultural validated [4, 5].

In morocco, colorectal cancer is very frequent with many published studies [6–8]. Previously a Moroccan version has been validated in both the Moroccan immigrant population in the Netherlands [9] and cancer patients in a national study [10] However, the proportion of colorectal cancer cases in the previous validation was limited to 8%, thereby possibly not representative enough as regards this type of cancer specifically known for its alterations on the different features of HRQOL; namely the physical (e.g. social limitations as a result of physical health, pain/discomfort, general health perception), social (e.g. distress management, inability to socialize) and medical aspects (e.g. diarrhea, fatigue, impaired body image, sexual problems) [11].

Our study aimed to examine the psychometric properties of the QLQ-C30 (3.0 version) questionnaire and assess its reliability and validity in patients followed for colorectal cancer in the National Oncology Institute of Rabat.

## Materials and methods

Our article is written following the STROBE (Strengthening the Reporting of Observational Studies in Epidemiology) directive guidelines for observational studies [12].

We conducted an observational cross-sectional study on colorectal cancer patients receiving treatment at the National Institute of Oncology of Morocco, in the period extending from February 2015 to June 2017. We retrospectively identified patients aged at least 18 years old with a histologically diagnosed primary colorectal cancer. All the patients who were on radiotherapy or chemotherapy and those with debilitating affections were excluded. Eligible patients were invited to participate by a personal direct approach in the waiting room at the outpatient clinic or after the consult. All information concerning the study was given to the participants and a signed written informed consent was required for recruitment. This study was approved by the local ethical committee of the Faculty of Medicine of Rabat (N° 79/2017).

The EORTC QLQ-C30 is a 30-item questionnaire with nine multi-item and six single-item scales, which reflects the multidimensionality of the quality-of-life construct [4]. The questionnaire includes five functional subscales (i.e., physical functioning, role functioning, emotional functioning, cognitive functioning, and social functioning), three symptom subscales (i.e., fatigue, nausea and vomiting, and pain), a global QoL subscale, and six single symptom items (i.e., dyspnea, insomnia, appetite loss, constipation, diarrhea, and financial difficulties). The two items of the global quality of life scale are scored on a 7 point Likert scale ranging from 1 (very poor) to 7 (excellent), while all other items are scored on a 4 point Likert scale ranging from 1 (not at all) to 4 (very much). According to the QLQ-C30 scoring manual [10], scores were standardized by linear transformation with results ranging from 0 to 100. Higher scores in functioning scales represent a higher level of functioning, while higher scores on symptom scales as well as single items reflect a greater level of impairment.

We used the original Moroccan version of EORTC QLQ-C30 (version 3.0) which was already translated to Moroccan Arabic according to the published guidelines for cross-cultural adaptation of health-related quality of life measurements in another national study. Words with no synonym in Moroccan Arabic were replaced by their equivalent in standard Arabic alongside a short explanatory description [10].

The sample size was determined according to the strainer curve [13]. A sample of 120 patients was required for an interclass correlation **(ICC)** of 0.70 and a precision of ±0.10. To characterize our sample, data on diagnosis, disease stage, treatment, and demographics were either retrieved from the hospital medical records or the patients using a predesigned form. The questionnaire was either self-administered or administered in the form of an interview for patients unable to complete it. Twenty-nine patients were invited to complete the questionnaire for a second time after 1 to 2 weeks to examine the test-retest reliability. Descriptive statistics were generated through mean, median and standard deviation to evaluate missing data, and score distributions.

The internal consistency of the multi-item scales was assessed by Cronbach’s alpha coefficient and a value of 0.70 or greater was considered adequate [14]. Furthermore, both convergent and discriminant validity were assessed. Known group validity was also evaluated by comparing the subgroups of patients with and without a stoma, as well as those with colon or rectal cancer locations using the Mann–Whitney U test.

Likewise, multitrait scaling analysis was also performed as in other EORTC QLQ-C30 transcultural validations and evaluation of other health status measures [4, 15]. This analysis method allowed the determination of item convergent validity, which is supported by a correlation above 0.40. On the other hand, discriminant validity confirmation is achieved by the demonstration of a higher correlation of the item with its own scale rather than the other scales. A definite scaling error was assumed if the correlation of an item with another scale exceeded the correlation with its own [16]. All statistical analyses were performed using SPSS version 18. A significant *P* value was considered if *p* < 0,05.

**Fig. 1:**
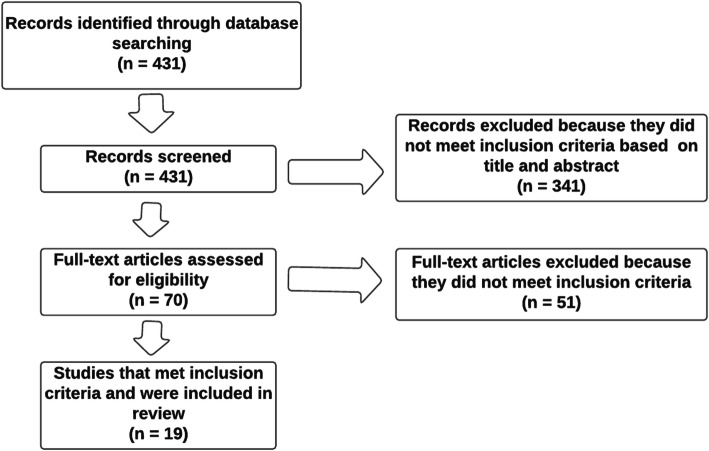
Flowchart of the literature review search on The EORTC QLQ-C30 validation studies

To compare the result of this study to other previous validation, We performed a systematic search of SpringerLink, PubMed, and ScienceDirect databases to identify studies about the EORTC-QLQ C30 validation. The MeSH-terms (Medical SubHeadings) used were: “Quality of Life” combined with “Colorectal Neoplasms/psychology”, “Colorectal Neoplasms/therapy”, “Psychometrics/methods” and the PROM’s name. (Fig. 1).

## Results

### Patient characteristics

#### Sociodemographic data

Our study included 120 patients with colorectal cancer among which 38 (32%) and 82 (68%) had colon and rectal cancer respectively. Twenty-nine patients (24%) had a stoma. The mean age of diagnosis was 54 years (+/− 3.3) (Range: 25–86). The clinical and sociodemographic characteristics of our patients are presented in Table 1.

**Table 1:** Demographic and clinical features of patients

*n* = 120	Number (%)
Age (median = 54; Range = 25–86)	
Gender	
Male	76 (63.3%)
Female	44 (36.7%)
Marital status	
Married	91(75.8%)
Single	12 (10.1%)
Other	17 (14.1%)
Educational status	
illiterate	58 (48.3%)
Primary and middle school	50 (41.6%)
High school degree	12 (10.1%)
Socioeconomic status	
Low	58 (48.3%)
intermediate	56 (46.6%)
Good	6 (5%)
Cancer Location	
Colon	38 (31.7%)
Rectum	82 (68.3%)
Stoma	
Yes	29 (24.2%)
No	91 (75.8%)
Chemoradiotherapy	
Yes	87 (72.5%)
No	33 (27.5%)
Medical insurance	
Yes	47 (39.1%)
No	73 (60.8%)

The average time to complete the questionnaire ranged from 9.6 to 17 min. Sixty-seven patients (56%) could not complete the questionnaire without the help of an interviewer. The patients considered the time of questionnaire administration after the medical consult as inappropriate. Patients reported difficulty differentiating questions 29 and 30 concerning the overall quality of life and general health.

The Moroccan QLQ-C30 version showed good internal consistency with an alpha Cronbach coefficient ≥ 0.70. Test-retest reliability was assessed using the intraclass correlation coefficient, which ranged from 0.78 for “fatigue” to 0.92 for “social functioning”. The lowest test-retest reliability coefficient was for Sleep loss (*r* = 0.78). (Table 2).

**Table 2:** Internal Consistency Coefficient

Scale	Cronbach’s alpha		
	overall	With stoma	Without stoma
Global health status / quality of life			
Global health status / quality of life	0,82	0.87	0.81
Functioning scales			
Physical	0.79	0.83	0.79
Role	0.82	0.88	0.81
Emotional	0.80	0.84	0.80
Cognitive	0.80	0.85	0.79
Social and Family	0.83	0.83	0.83
Multi-item symptoms scales			
Fatigue	0,78	0.83	0,76
Nausea/Vomiting	0.83	0.86	0.82
Pain	0.79	0.83	0.79

### Convergent and discriminant validity

All questionnaire items fulfilled the criteria for convergent and discriminant validity exceeding r ≥ 0.40, except for question number 5 (Q5). The correlation of the fifth question with its own scale (the physical functioning scale) was r = 0.395, therefore below the minimum required correlation coefficient. (Table 3).

**Table 3:** Multitrait scaling analysis

	GHS/QOL	PFS	ROS	COGS	EMOS	SOCS	FATIGS	NVS	PAINS
Q29	,913^**^	,429^**^	,265^**^	,216^*^	,193^*^	,216^*^	-,323	-,072	-,267
Q30	,932^**^	,351^**^	,337^**^	,169	,111	,310^**^	-,243^**^	-,044	-,223
Q1	-,298^**^	-,738^**^	-,320^**^	-,367^**^	-,207^*^	-,009	,422^**^	,056	,361
Q2	-,297^**^	-,784^**^	-,507^**^	-,381^**^	-,321^**^	-,223^*^	,562^**^	,220	,368
Q3	-,259^**^	-,732^**^	-,295^**^	-,358^**^	-,212^*^	-,184^*^	,332^**^	,264	,243
Q4	-,334^**^	-,718^**^	-,485^**^	-,486^**^	-,354^**^	-,265^**^	,554^**^	,362	,402
Q5	-,274^**^	-, 395	-,325^**^	-,189^*^	-,002	-,121	,134	,200	,135
Q6	-,354^**^	-,527^**^	-,926^**^	-,247^**^	-,226^*^	-,206^*^	,474^**^	,284	,303
Q7	-,251^**^	-,507^**^	-,921^**^	-,223^*^	-,170	-,192^*^	,469^**^	,346^**^	,261
Q20	-,235^**^	-,537^**^	-,217^*^	-,869^**^	-,444^**^	-,235^**^	,453^**^	,356^**^	,457
Q25	-,113	-,342^**^	-,219^*^	-,840^**^	-,438^**^	-,265^**^	,372^**^	,325^**^	,294
Q21	-,112	-,273^**^	-,130	-,404^**^	-,901^**^	-,146	,553^**^	,309^**^	,511
Q22	-,216^*^	-,260^**^	-,140	-,397^**^	-,855^**^	-,192^*^	,527^**^	,238^**^	,411
Q23	-,171	-,350^**^	-,193^*^	-,495^**^	-,856^**^	-,188^*^	,641^**^	,280^**^	,558
Q24	-,041	-,224^*^	-,283^**^	-,464^**^	-,775^**^	-,221^*^	,546^**^	,486^**^	,470
Q26	-,203^*^	-,181^*^	-,167	-,253^**^	-,173	-,916^**^	,080	,147	,071
Q27	-,325^**^	-,239^**^	-,227^*^	-,278^**^	-,225^*^	-,902^**^	,126	,273^**^	,145
Q10	-,306^**^	-,586^**^	-,412^**^	-,429^**^	-,518^**^	-,094	,796^**^	,315^**^	,559
Q12	-,200^*^	-,414^**^	-,357^**^	-,431^**^	-,590^**^	,007	,850^**^	,396^**^	,560
Q18	-,260^**^	-,488^**^	-,508^**^	-,351^**^	-,555^**^	-,196^*^	,850^**^	,347^**^	,549
Q14	-,021	-,280^**^	-,379^**^	-,306^**^	-,393^**^	-,166	,417^**^	,886^**^	,249
Q15	-,089	-,271^**^	-,219^*^	-,399^**^	-,276^**^	-,238^**^	,332^**^	,879^**^	,143
Q9	-,228^*^	-,443^**^	-,182^*^	-,363^**^	-,381^**^	-,116	,530^**^	,137	,871^**^
Q19	-,233^*^	-,335^**^	-,351^**^	-,410^**^	-,619^**^	-,090	,636^**^	,251^**^	,876^**^

*GHS/QOL* (Global health status & quality of life), *PFS* (physical functioning scale), *ROS* (role functioning scale), *COGS* (cognitive functioning), *EMOS* (emotional functioning scale), *SOCS* (social functioning scale), *FATIGS* (fatigue symptom), *NVS* (nausea & vomiting symptom), *PAINS* (pain symptom)

The comparison of clinically distinct patient groups, namely patients with and without stoma reported a high symptom score for diarrhea (*p* = 0.015) and social functioning (*p* = 0.038) for patients with a stoma. (Table 4) On the other hand, when comparing patients with colon or rectal cancer, the latter reported statistically significant bad global health status and quality of life (*p* = 0.016) and emotional functioning (*p* = 0.028). (Table 5).

**Table 4:** Group comparison between patients with and without a stoma

	Stoma	No Stoma	*P*- value
	Mean (SD)	Mean (SD)	
Global health status & quality of life	58,2 (19,8)	63,4 (19,6)	0.356
Physical Functioning	65,4 (27,3)	73,4 (18,8)	0.145
Role functioning	45,6 (33,4)	55,9 (38,4)	0.188
Emotional functioning	74,2 (23,7)	78,1 (24,7)	0.333
Cognitive functioning	83,3 (26,5)	89,9 (21,7)	0.182
Social Functioning	72,2 (29,6)	82,9 (26,8)	**0.038**
Fatigue	64,8 (31,3)	68,6 (34,3)	0.423
Nausea & vomiting	13,8 (21,1)	9,4 (18,3)	0.173
Pain	36,1 (32,4)	40,8 (33,3)	0.744
Dyspnea	30,5 (41,3)	20,2 (29,1)	0.456
Insomnia	47,2 (41,3)	40,5 (39,6)	0.450
Appetite loss	36,1 (36,1)	26,1 (32,8)	0.081
Constipation	39.1 (37.2)	28.6 (33.6)	0.182
Diarrhea	14.9 (21.1)	32.9 (36.3)	**0.015**
Financial difficulty	62,1 (39,8)	60,7 (38,7)	0.783

**Table 5:** Group comparison between patients with colon and rectum cancer

	Colon (*n* = 38)	Rectum (*n* = 82)	*P* value
	Mean (SD)	Mean (SD)	
Global health status & quality of life	55,0 (20,7)	64,3 (18,6)	**0.016**
Physical Functioning	71,9 (23,4)	71,4 (20,1)	0.911
Role functioning	51,3 (38,0)	54,6 (33,7)	0.627
Emotional functioning	82,4 (20,3)	71,4 (28,6)	**0.028**
Cognitive functioning	91,2 (16,7)	86,1 (22,1)	0.211
Social Functioning	76,3 (29,6)	82,5 (26,5)	0.254
Fatigue	29,5 (24,9)	40,1 (28,9)	0.248
Nausea & vomiting	5,2 (13,4)	10,3 (20,0)	0.157
Pain	25,4 (26,7)	31,3 (32,3)	0.332
Dyspnea	13,1 (23,9)	23,1 (29,9)	0.072
Insomnia	33,3 (31,9)	33,7 (37,9)	0.954
Appetite loss	21,9 (30,2)	24,3 (32,3)	0.693
Constipation	35,0 (35,4)	29,2 (34,1)	0.392
Diarrhea	25,4 (35,0)	30,0 (33,7)	0.490
Financial difficulty	51,7 (41,5)	63,4 (33,7)	0.105

## Discussion

This study aimed to examine the psychometric properties of the Moroccan version of the EORTC QLQ-C30 questionnaire in colorectal cancer patients, which showed good psychometric properties in colorectal cancer patients.

Although a high rate of illiteracy in the Moroccan population, the average time required to complete the questionnaire varied from 9.6 to 17 min, either with or without the help of the interviewer. As this average response time is similar to those reported in other validation studies, the Moroccan version can be adequately used in other clinical studies [17, 18].

The assessment of the psychometric properties of the EORTC QLQ-C30 was according to the methodology used in the other validation studies. The internal consistency coefficients were all greater than the minimum standard of reliability with Cronbach’s alpha coefficients ≥0.78 (Table 6).

**Table 6:** Scale construction of EORTC QLQ-C30 items internal consistency comparison

Data	Sample (n)	PF	RF	CF	EF	SF	QL	FS	PS	NVS
Our study	120 (CRC)	0.79	0.82	0.80	0.80	0.83	0,82	0,78	0.79	0.83
Moroccan (Nejjari)	125 (HC)	0.84	0.94	0.34	0.89	0.80	0.87	0.83	0.78	0.74
Lebanese	200 (HC)	0.89	0.80	0.60	0.79	0.65	0.89	0.81	0.75	0.43
United Arab Emirates	87 (BC)	0.76	0.84	0.67	0.87	0.79	0.86	0.84	0.50	0.86
Malysian chinese	96 (CRC)	0.74	0.92	0.26(	0.79	0.71	0.91	0.79	0.77	0.82
American	489 (NCP)	0.77	0.88	0.58	0.84	0.80	0.84	0.81	0.83	0.50
Turkish	194 (LC)	0.70	0.89	0.71	0.84	0.76	0.94	0.94	0.87	0.76
German	1864 (HC)	0.57	0.36	0.85	0.72	0.78	0.90	0.86	0.89	0.75
Ethiopian Amharic	153 (GC)	0.83	–	0.29	0.93	0.82	0.92	0.89	0.73	0.75
Brazilian portugese	986 (LC)	0.80	0.79	0.57	0.84	0.69	0.78	0.78	0.81	0.68
Iranian	132 (BC)	0.76	0.77	0.77	0.77	0.73	0.82	0.65	0.66	0.69
indonesian	128 (HC)	0.82	0.79	0.82	0.78	0.83	0.80	0.72	0.85	0.70
korean	170 (NC)	0.87	0.87	0.60	0.86	0.82	0.84	0.78	0.84	0.85
Spanish mexican	150 (GRC)	0.79	0.8	0.32	0.85	0.8	0.9	0.78	0.55	0.8
Taiwan chinese	99 (LC)	0.85	0.92	0.58	0.81	0.82	0.86	0.80	0.81	0.74
Thailand	(NC)	0.75	0.76	0.50	0.86	0.63	0.90	0.73	0.80	0.82

***CRC*** **(colorectal cancer),** ***HC*** **(heterogenous cancers),** ***BC*** **(breast cancer),** ***NCP*** **(non cancerous population),** ***LC*** **(lung cancer),** ***GC*** **(gynecological cancer),*GRC*** **(gastric cancer)**

In the original Moroccan validation as well as in other Arabic versions, namely the Lebanese [19] and the UAE [20], problems have been reported with the psychometric properties of the “cognitive functioning” scale. This could be explained by the fact that the scales containing few items are prone to achieve the lowest coefficients [21]. Other studies justified the low internal consistency for this scale by the fact that the concentration and memory items could be affected by other elements such as pain and fatigue [21, 22].

The different validation studies were either conducted on populations of non-cancer-related diseases, heterogeneous malignancies, or specific cancers such as colorectal, lung, breast, or other gynecologic neoplasms. The internal consistency coefficients were overall acceptable despite the type of addressed disease. (Table 6) Only one other EORTC QLQ-C30 validation was based on colorectal cancer patients, showing low internal consistency for cognitive and social/family functioning [21].

The EORTC QLQ-C30 was reported sensitive to the differences in clinical status and clinical change over time [22]. In our study, the comparison of the subgroups demonstrated the ability of the scales/items to differentiate between clinically distinct patient groups. In fact, the EORTC QLQ-C30 allowed differentiating between patients with and without a stoma, thereby showing higher symptom scores for diarrhea (*p* = 0.013) and financial difficulty (*P* = 0.035) in patients with a stoma. Similarly, the Chinese-Malaysian study reported high symptom scores on the financial difficulty scale, higher impairment in physical and social/family functioning, and lower constipation symptoms [21]. In fact, the impairment of quality of life for patients with and without stoma has been challenged in many studies which either proved no difference or a better quality of lif e[23].

On the other hand, patients with rectal cancer showed statistically significant bad Global health status and quality of life, low emotional functioning, and higher fatigue symptoms compared to patients with colon cancer. Rectal cancer patients on the other hand were more likely to have an altered HRQOL compared to colon malignancies in the other validation studies [24].

Multitrait scaling analysis was conducted in the majority of validation studies to prove convergent and discriminant validity. Regarding this analytic tool, the Moroccan version of the QLQ-C30 met the criteria for validity with item-scale correlation > 0.40, except for the number 5 question “need help in eating/ dressing/washing” which is slightly inferior to the minimum correlation with its own scale (physical functioning). However, in comparison to other scales, this item demonstrates a higher correlation with its scale with r = 0.395. This item is considered the most unstable component of the EORTC QLQ-C30 questionnaire as it not only lacks convergent and discriminant validity in other validation studies [9], but also shows a correlation with the emotional functioning scale [25].

### Study limitations

The use of multiscaling analysis to test convergent and divergent reliability might be argued as the item-scale correlation might be a function of shared trait variance or shared error variance. Although a more advanced technique such as confirmatory factor analysis might be suggested, the multiscaling analysis remains the most used method in the other transcultural validations of the EORTC QLQ-C30.

Another possible limitation for this study is the small sample size of cancer patients in each disease subgroup and cancer location (rectum and colon), which makes subgroup analyses difficult. Furthermore, the majority of patients recruited came from outpatient rather than inpatient units, limiting the assessment of responsiveness over time.

### Study strengths

The EORTC QLQ-C30 demonstrates a good validity in Moroccan colorectal cancer patients which could encourage its use in future clinical trials assessment, as well as for the comparison with other HRQOL questionnaires specific to colorectal cancer, namely the EORTC QLQ-C29. The results of this study provide valuable data for comparing the HRQOL of colorectal cancer patients.

## Conclusion

In conclusion, the findings from our study demonstrate that the Moroccan version of the EORTC QLQ-C30 is valid and reliable for HRQOL assessment in patients with colorectal cancer. The EORTC developed another disease-specific questionnaire for colorectal cancer patients, namely the EORTC QLQ-C29 which requires further exploration for potential use in routine clinical practice.

## Supplementary Information

**Additional file 1.**

## Data Availability

All questionnaires and consent forms are available at The National Institute of oncology of Rabat, where the study was conducted. The data was pooled and analysed at The Laboratory of Clinical Research and Epidemiology of Mohammed V University.

## Abbreviations

EORTC: European organization for research and treatment of Cancer
QLQ: Quality of Life Questionnaire
