# Supplementary material for: Psychometric validation of the Moroccan version of the EORTC QLQ-C30 in colorectal Cancer patients: cross-sectional study and systematic literature review
Source: BMC Cancer. 2021 Jan 27;21:99. doi: 10.1186/s12885-021-07793-w (PMC7839186; doi:10.1186/s12885-021-07793-w)
Supplement: Supplementary file 1 — Additional file 1. [file 12885_2021_7793_MOESM1_ESM.docx]

# supplementary files :

**Appendix 1 : Arabic version of the EORTC QLQ-C30 questionnaire**

**تايخصنا نعرفو شي حوايج عليك و على صحتك. من فضلك جاوب على هاد الأسئلة و اختار الجواب اللي يناسبك. ما كاينش شي جواب صحيح أو غالط. المعلومات اللي غادي تعطي غادي تبقى سرية.**

- سميتك:

- تاريخ الازدياد ( النهار, الشهر, العام ):

- تاريخ ديال اليوم ( النهار, الشهر, العام ):

1 - واش تايجيك شي مشكل مني تادير شي خدمة تاتعيّ بحال تهزشي شانطة أو قفة تقيلة.

- نهائيا
- غير شويا
- مرة مرة
- بزاف

2 - واش تاتعيا فاش تاتمشا بزاف

- نهائيا
- غير شويا
- مرة مرة
- بزاف

3 - واش تاتعيا فاش تاتمشا هي شويا خارج الدار

- نهائيا
- غير شويا
- مرة مرة
- بزاف

4 - واش تاتحتاج تبقا فالفراش أو تكلس فاش كاتكون فالدار

- نهائيا
- غير شويا
- مرة مرة
- بزاف

5 - واش تاتحتاج شي واحد يعاونك فالماكلا، فالباس، فالغسيل، باش تمشي لمرحاض ( بيت الما )

- نهائيا
- غير شويا
- مرة مرة
- بزاف

**فالسيمانا اللي فاتت**

6 - واش حسيتي براسك ما قادرش دير الخدمة ديالك أو الشغالات ديال كل نهار

- نهائيا
- غير شويا
- مرة مرة
- بزاف

7 - واش حسيتي براسك ما قادرش ديرالهوايات ديالك

- نهائيا
- غير شويا
- مرة مرة
- بزاف

8 - واش جاك ضيق فالتنفس ( النهجة، القذفة )

- نهائيا
- غير شويا
- مرة مرة
- بزاف

9 - واش جاك لحريق

- نهائيا
- غير شويا
- مرة مرة
- بزاف

10 - واش حتاجيتي ترتاح

- نهائيا
- غير شويا
- مرة مرة
- بزاف

11 - واش عندك شي مشكل فالنعاس

- نهائيا
- غير شويا
- مرة مرة
- بزاف

12 - واش كنتي حاس براسك مرخي ( ضعيف)

- نهائيا
- غير شويا
- مرة مرة
- بزاف

13 - واش نقصاتلك الشهية

- نهائيا
- غير شويا
- مرة مرة
- بزاف

14 - واش تتحس بترويعا

- نهائيا
- غير شويا
- مرة مرة
- بزاف

15 - واش تقييتي (رديتي)

- نهائيا
- غير شويا
- مرة مرة
- بزاف

16 - واش كنتي مقبوط (معصوم)

- نهائيا
- غير شويا
- مرة مرة
- بزاف

فالسيمانا لي فاتت

17 - واش كانت كرشك جارية (طايحة عليك الكرش)

- نهائيا
- غير شويا
- مرة مرة
- بزاف

18 - واش كنتي عيان

- نهائيا
- غير شويا
- مرة مرة
- بزاف

19 - واش لحريق كان كيأثرعلى الشغالات ديالك ديال كلا نهار

- نهائيا
- غير شويا
- مرة مرة
- بزاف

20 - واش جاك شي مشكل فالتركيز فاش كاتقرى شي جورنال أو فاش كتفرج فالتيليفيزيون

- نهائيا
- غير شويا
- مرة مرة
- بزاف

21 - واش حسيتي براسك معصب

- نهائيا
- غير شويا
- مرة مرة
- بزاف

22 - واش حسيتي براسك مقلق

- نهائيا
- غير شويا
- مرة مرة
- بزاف

23 - واش حسيتي براسك منفعل (كتقلق دغيا )

- نهائيا
- غير شويا
- مرة مرة
- بزاف

24 - واش حسيتي براسك مكتئب (مغموم )

- نهائيا
- غير شويا
- مرة مرة
- بزاف

25 - واش كان عندك مشكل تتفكر شي حوايج (مشكل نسيان )

- نهائيا
- غير شويا
- مرة مرة
- بزاف

26 - واش هاد المرض ديالك أو الدوا اللي تاتخد أثر على علاقتك مع العائلة

- نهائيا
- غير شويا
- مرة مرة
- بزاف

27 - واش هاد المرض ديالك أو الدوا اللي تاتخد أثر على علاقتك مع الناس

- نهائيا
- غير شويا
- مرة مرة
- بزاف

28 - واش هاد المرض ديالك أو الدوا اللي تاتخد سبب ليك شي مشاكل فالمصروف (مشاكل مادية )

- نهائيا
- غير شويا
- مرة مرة
- بزاف

**بالنسبة للأسئلة اللي جايا اختار الجواب اللي يناسبك من 1 إلى 7**

29 - شحال تقيم ( تعطي ) صحتك فالسيمانا اللي فاتت

ممتاز(7) ضعيف (1)

30 - شحال تقيم ( تعطي ) الجودة ديال حياتك فالسيمانا اللي فاتت

ممتاز(7) ضعيف (1)

**Appendix 2 : English version of the EORTC QLQ-C30 questionnaire**

**We are interested in some things about you and your health. Please answer all of the questions yourself by circling the number that best applies to you. There are no "right" or "wrong" answers. The information that you provide will remain strictly confidential.**

Please fill in your initials:

Your birthdate (Day, Month, Year):

Today's date (Day, Month, Year):

1. Do you have any trouble doing strenuous activities, like carrying a heavy shopping bag or a suitcase?

- Not at All
- A Little
- Quite a Bit
- Very Much

2. Do you have any trouble taking a long walk?

- Not at All
- A Little
- Quite a Bit
- Very Much

3. Do you have any trouble taking a short walk outside of the house?

- Not at All
- A Little
- Quite a Bit
- Very Much

4. Do you need to stay in bed or a chair during the day?

- Not at All
- A Little
- Quite a Bit
- Very Much

5. Do you need help with eating, dressing, washing yourself or using the toilet?

- Not at All
- A Little
- Quite a Bit
- Very Much

6. Were you limited in doing either your work or other daily activities?

- Not at All
- A Little
- Quite a Bit
- Very Much

7. Were you limited in pursuing your hobbies or other leisure time activities?

- Not at All
- A Little
- Quite a Bit
- Very Much

8. Were you short of breath?

- Not at All
- A Little
- Quite a Bit
- Very Much

9. Have you had pain?

- Not at All
- A Little
- Quite a Bit
- Very Much

10. Did you need to rest?

- Not at All
- A Little
- Quite a Bit
- Very Much

11. Have you had trouble sleeping?

- Not at All
- A Little
- Quite a Bit
- Very Much

12. Have you felt weak?

- Not at All
- A Little
- Quite a Bit
- Very Much

13. Have you lacked appetite?

- Not at All
- A Little
- Quite a Bit
- Very Much

14. Have you felt nauseated?

- Not at All
- A Little
- Quite a Bit
- Very Much

15. Have you vomited?

- Not at All
- A Little
- Quite a Bit
- Very Much

16. Have you been constipated?

- Not at All
- A Little
- Quite a Bit
- Very Much

**During the past week:**

17. Have you had diarrhea?

- Not at All
- A Little
- Quite a Bit
- Very Much

18. Were you tired?

- Not at All
- A Little
- Quite a Bit
- Very Much

19. Did pain interfere with your daily activities? Not at All

- A Little
- Quite a Bit
- Very Much

20. Have you had difficulty in concentrating on things, like reading a newspaper or watching television?

- Not at All
- A Little
- Quite a Bit
- Very Much

21. Did you feel tense?

- Not at All
- A Little
- Quite a Bit
- Very Much

22. Did you worry?

- Not at All
- A Little
- Quite a Bit
- Very Much

23. Did you feel irritable?

- Not at All
- A Little
- Quite a Bit
- Very Much

24. Did you feel depressed?

- Not at All
- A Little
- Quite a Bit
- Very Much

25. Have you had difficulty remembering things?

- Not at All
- A Little
- Quite a Bit
- Very Much

26. Has your physical condition or medical treatment interfered with your family life?

- Not at All
- A Little
- Quite a Bit
- Very Much

27. Has your physical condition or medical treatment interfered with your social activities?

- Not at All
- A Little
- Quite a Bit
- Very Much

28. Has your physical condition or medical treatment caused you financial difficulties?

- Not at All
- A Little
- Quite a Bit
- Very Much

**For the following questions please circle the number between 1 and 7 that best applies to you :**

29. How would you rate your overall health during the past week?

Very poor (1) Excellent (7)

30. How would you rate your overall quality of life during the past week?

Very poor (1) Excellent (7)
